# On the Treatment of Eczema by Bantingism

**Published:** 1882-09-20

**Authors:** Balmanno Squire

**Affiliations:** London


### ON THE TREATMENT OF ECZEMA BY BANTINGISM.

BY BALMANNO SQUIRE, M. B., LOND.

It is familiar to every practitioner that eczema is specially common amongst infants, and particularly amongst lymphatic infants; that is to say, fat and pasty-looking infants. I do not refer only to those instances in which the very fatness of the infant is the mechanical cause of the complaint; that is to say, where a "fold" of skin in the fat infant becomes raw and discharging (intertrigo); but I refer to the well known fact that infants of this constitution are more liable than others to eczema (of the scalp and other parts), not coming under the head of "intertrigo," and that their eczema is more profuse in its discharge, whether that discharge be serous or purulent, than it is in other infants; it is also more obstinate

I recently recorded in the Journal some experiments I had made with iodoform as an application in such cases, and which succeeded very well as a means of reducing the eczema of such infants from the discharging to the dry condition pretty rapidly. As to that, I was supported by other writers.

But from some observations I have recently made, I have reason to think that Bantingism as applied to such infants is as rapid in its effects, and, if sustained, is of more permanent efficacy. Some years ago the late Mr. Banting was under my care for eczema, and I had an opportunity of conversing with him pretty frequently on the system of which he was the apostle. It is through this accident that the idea suggested itself to me.

I have used the word infant for the sake of convenience, but I refer rather to very young children. To Bantingise a suckling infant is, of course, not very practicable, but a child of two or three years old can be dieted very readily. It does not appear necessary to the end in view that the diet should be restricted in quantity as well as in quality, which, of course, was essential under the *regime* laid down by Mr. Banting, or rather by the late Mr. Harvey (the aurist) for him. It is simply necessary to limit the fat-producing elements of food for the amelioration of eczema in lymphatic young children. At least, so I have found. By this means their excessive obesity becomes diminished and their eczema very remarkably improved within ten days of commencing the regimen, and that without any injury whatever to their general health, so far as I can judge.

In place of pure milk, they should take milk diluted with an equal or even a double quantity of water. In place of bread and butter they should take dry toast or dry biscuits; and with these particular articles of food they may be supplied indefinitely. All the fat is to be carefully cut away from such meat as they may partake of, and they should not be allowed pork, veal or lamb. They may have poultry or game or fish, except the oily kind of fishes, such as herrings, salmon, eels, etc.; and the fish they partake of should be broiled (not fried). They may eat boiled vegetable tops, but not vegetable roots, such as potato, parsnip, beetroot, turnip or carrot. Beef-tea (the melted fat being carefully skimmed off) is permissible in any quantity, and so also toast and water. Cooked fruit (not sweetened) may also be allowed.

But the principles of the Banting treatment are universally known. It only remains for me to say that cod-liver oil (the favorite remedy, *par excellence*, for the condition of which I am speaking) is quite incompatible with the treatment I am advocating. Of course, the health of the child should be watched; and it should be weighed at the commencement of its dieting, and afterward from week to week.—*British Medical Journal, April 8, 1882, p. 499.*

